# Machine Learning Approach with Harmonized Multinational Datasets for Enhanced Prediction of Hypothyroidism in Patients with Type 2 Diabetes

**DOI:** 10.3390/diagnostics14111152

**Published:** 2024-05-31

**Authors:** Robert P. Adelson, Anurag Garikipati, Yunfan Zhou, Madalina Ciobanu, Ken Tawara, Gina Barnes, Navan Preet Singh, Qingqing Mao, Ritankar Das

**Affiliations:** Montera, Inc. dba Forta, 548 Market St, PMB 89605, San Francisco, CA 94104-5401, USA; robert.adelson@fortahealth.com (R.P.A.); agarikipati@fortahealth.com (A.G.); reesezhou88@gmail.com (Y.Z.); mciobanu@fortahealth.com (M.C.); ken.tawara@fortahealth.com (K.T.); gbarnes@fortahealth.com (G.B.); nsingh@fortahealth.com (N.P.S.); ritankar@fortahealth.com (R.D.)

**Keywords:** machine learning, decision support, hypothyroidism, type 2 diabetes

## Abstract

Type 2 diabetes (T2D) is a global health concern with increasing prevalence. Comorbid hypothyroidism (HT) exacerbates kidney, cardiac, neurological and other complications of T2D; these risks can be mitigated pharmacologically upon detecting HT. The current HT standard of care (SOC) screening in T2D is infrequent, delaying HT diagnosis and treatment. We present a first-to-date machine learning algorithm (MLA) clinical decision tool to classify patients as low vs. high risk for developing HT comorbid with T2D; the MLA was developed using readily available patient data from harmonized multinational datasets. The MLA was trained on data from NIH All of US (AoU) and UK Biobank (UKBB) (Combined dataset) and achieved a high negative predictive value (NPV) of 0.989 and an AUROC of 0.762 in the Combined dataset, exceeding AUROCs for the models trained on AoU or UKBB alone (0.666 and 0.622, respectively), indicating that increasing dataset diversity for MLA training improves performance. This high-NPV automated tool can supplement SOC screening and rule out T2D patients with low HT risk, allowing for the prioritization of lab-based testing for at-risk patients. Conversely, an MLA output that designates a patient to be at risk of developing HT allows for tailored clinical management and thereby promotes improved patient outcomes.

## 1. Introduction

The prevalence of diabetes in the United States (US) among adults is estimated to be >14%, impacting approximately 38.1 million individuals; type 2 diabetes (T2D) accounts for 90–95% of these cases [1,2]. This number is expected to rise in the US to 39.7 million by 2030, and further increase to 60.6 million by 2060 [3]. Similar trends in growth are expected globally, with the number of individuals with T2D increasing from 537 million [4] to a projected 784 million by 2045 [5]. Financial costs associated with T2D total approximately USD 327 billion in the US, with comorbid conditions increasing costs by USD 9000 per patient within the first year of diagnosis [2,6]. One such T2D comorbidity is hypothyroidism (HT), a metabolic disorder that dysregulates glucose and insulin homeostasis [7,8] which can exacerbate diabetic complications and thereby lead to poorer patient outcomes and increased care costs [7,9]. In the US, the financial impact of HT reaches USD 2.1 billion/year, encompassing healthcare utilization, productivity loss, and costs related to managing HT [10,11,12]. HT occurs in about 12% of adults with T2D [13,14] which is similar to the estimated 11.7% HT prevalence in the general adult population [12]. Individuals with T2D have a 1.93-fold increased risk of developing subclinical HT by comparison to the general population [15,16], which can progress to overt HT at a rate of up to 6% per year for individuals without diabetes, and >11% for individuals with diabetes [15,17,18,19,20]. Thus, the early identification and treatment of individuals with T2D who are at risk of developing HT are crucial to improve patient outcomes and minimize costs.

It is estimated that more than 6% of T2D patients have undiagnosed HT [11] and T2D and HT can present with overlapping symptoms, which complicates diagnosis [21]. T2D and HT each pose separate risks; however, when these two disorders co-occur, individuals can experience negative health outcomes specific to each disorder, or from the interaction between the two disorders. These outcomes include increased risk of cardiovascular disease, serious diabetic complications (e.g., vascular dysfunction, chronic kidney disease, etc.), depression, dementia, and in rare cases, myxedema, an extreme and life-threatening complication of untreated HT [15,16,20,22,23,24,25].

Stratifying patients with T2D who have HT or are at risk of developing HT may reduce disease morbidity by guiding clinical management strategies for at-risk patients, for example, by modifying and enhancing the monitoring of therapeutic medication for T2D [26]; however, the standard of care (SOC) screening guidelines for HT indicate infrequent testing (every 5 years in the absence of symptoms); they are additionally outdated and poorly aligned with screening recommendations noted in research on individuals with T2D [10,21,22,27,28,29]. Further, separate TSH screening recommendations for patients with T2D do not exist, which may be the result of underestimating T2D and HT interactions and their impact on overall health risks and demonstrates a gap in care for these individuals [15,21].

Assessment complexity may be alleviated with a more comprehensive screening approach that is data-driven and automated, and solely requires electronic health record (EHR) data to assess risks. Machine learning (ML), with its ability to analyze complex patient information, can offer the solution of delivering highly individualized predictions regarding HT risk in T2D patients.

### Related Work

ML and artificial intelligence (AI)-based clinical decision support (CDS) tools have recently gained widespread attention due to their potential for reducing costs, improving diagnostic reliability, nearly unlimited scalability, and ability to provide better treatment response predictions in complex disease management [30,31,32,33,34,35,36,37,38,39,40]. In T2D, ML-based tools improved blood glucose management and insulin dosage [41], and T2D detection and diagnosis [42,43,44,45], which, in turn, led to enhanced quality of care for individuals with diabetes [41]. However, there are no validated prediction tools to date to provide a clinical assessment of a T2D patient’s risk of developing HT. The previous limited research has explored ML-based risk prediction of HT in individuals with comorbidities (other than T2D) and for diagnostic classification. To predict HT complications caused by radiotherapy in head and neck cancer patients, Lee et al. evaluated the performance of three ML models and achieved area under the receiver operating characteristic curve (AUROC) values of 0.692–0.827 [46]. Naeem et al. evaluated patient clinical, demographic, and laboratory data using *k*-nearest neighbor (KNN), Naïve Bayes, and support vector machine (SVM) ML models to identify patients with HT; the SVM model yielded the best performance (accuracy: 84.72%) [47]. Rad et al. used ML to evaluate clinical symptoms of HT to classify patients as either having or not having the condition and achieved accuracies ranging from 0.64 to 0.83; in the best-performing model (random forest), the top three clinical features that contributed most to classification output were tiredness, cold extremities, and numb hands [48]. As reflected by the absence of research on ML-based prediction of HT for individuals with T2D, it is apparent that this remains an unmet need to address a condition that goes under-recognized in T2D patients, and can thereby lead to serious adverse health consequences for T2D patients [7,18,21,22,49,50]. 

**Figure 1:**
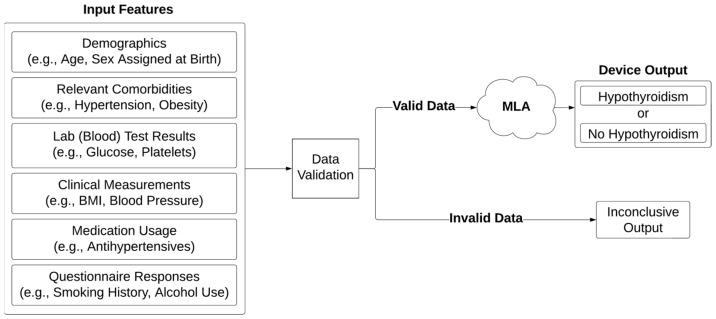
Diagram of inputs and outputs for each machine learning algorithm (MLA).

Here, we describe the development and validation of a first-to-date machine learning algorithm (MLA) for the prediction of a T2D patient’s likelihood of developing HT using two demographically distinct datasets and readily available input features (e.g., demographics, lab [blood] tests, and clinical measurements) (Figure 1). Datasets were individually used for training and testing ML models, and after being combined and harmonized, were used for developing an additional MLA (i.e., Combined MLA). We hypothesized that the use of the latter, Combined dataset would lead to enhanced MLA performance when compared to the performance of the models developed and validated on each of the datasets (prior to combining them). The MLA results are designed for easy interpretation by clinicians, with the output indicating HT risk, or with an inconclusive output in the case of invalid data inputs. With a negative predictive value (NPV) of 0.987 (95% confidence interval [CI]: 0.983–0.991), our MLA can effectively screen out T2D patients with low HT risk or decreased likelihood of developing HT, thereby prompting further clinical investigations for those who have a high HT risk. This MLA has the potential to significantly enhance clinical decision-making by offering a more consistent and simplified approach for identifying T2D individuals with HT risk.

## 2. Materials and Methods

### 2.1. Dataset

Our analysis was conducted using retrospective data from two publicly available datasets: the United Kingdom (UK) Biobank (UKBB) and the National Institutes of Health (NIH) All of Us (AoU). The UKBB contains biomedical data, including genetic and health information, for 500,000 UK participants [51]. The AoU Program currently has data from over 500,000 US individuals, including participants from diverse backgrounds, such as various marginalized groups [52]. Both datasets were delivered with de-identified data, ensuring HIPAA compliance; thus, this research is not considered human subjects research according to 45 US CFR 46.102. 

**Figure 2:**
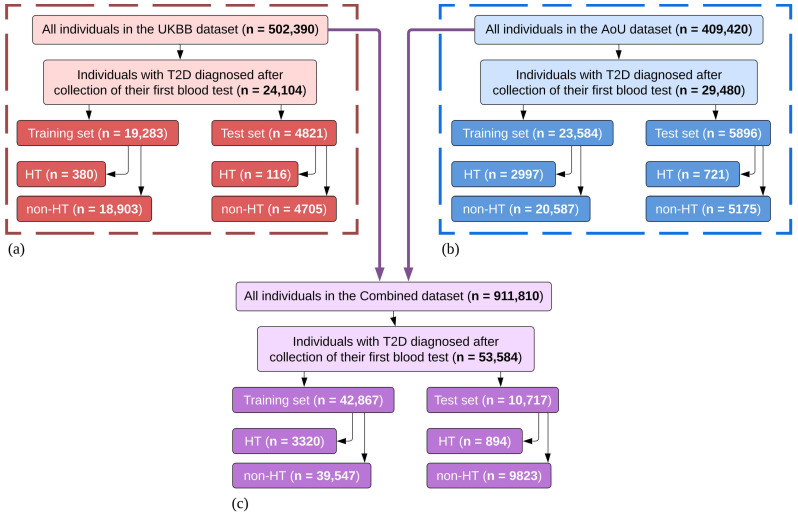
An attrition chart for the datasets used for modeling. (**a**) UK Biobank (UKBB) dataset. (**b**) All of Us (AoU) dataset. (**c**) Combined dataset. All individuals in the filtered datasets have a type 2 diabetes (T2D) diagnosis. Note: hypothyroidism (HT) and non-HT populations in the Combined training and test sets do not equal the sum of HT and non-HT populations in the training and test sets from UKBB and AoU datasets, respectively, due to a new train-test split being performed on the Combined dataset.

Data were filtered to select individuals who received a T2D diagnosis after the first lab (blood) test recorded in the datasets, as shown in Figure 2. It was required for the data to have biosample and health information, and <25% missingness of phenotypic data for any given individual. Diagnoses of T2D and HT were reported by health care providers (HCPs), and an HT diagnosis was considered the ground truth to identify the positive class of individuals. The T2D patients filtered from the UKBB dataset (*n* = 24,104) were combined with those filtered from the AoU dataset (*n* = 29,480) to yield a multinational Combined dataset (*n* = 53,584) with 4214 individuals diagnosed with HT (HT cohort, Combined dataset) and 49,370 individuals without an HT diagnosis (non-HT cohort, Combined dataset). For each of the three datasets (Combined, UKBB, AoU), a random selection of 20% of the T2D patients was used as a hold-out test set (i.e., test set), and the remaining 80% of the T2D patients formed the corresponding training set, as detailed in Figure 2. Each test set served to validate the performance of the corresponding model post-training. Each test set remained completely independent of the training process and was solely used to evaluate corresponding model results to determine the algorithm’s efficacy. This separation was maintained between the test set and the training set to prevent data leakage from the training set from inadvertently altering the performance of the MLA on the test set. Demographic information is shown in Table 1 for all training sets, and in the Supplemental information (Supplemental Table S1) for all test sets. 

### 2.2. Data Processing and Feature Selection

#### 2.2.1. Data Processing and Feature Selection for the UKBB Dataset

The UKBB dataset was downloaded with data on 502,390 individuals, with each individual having 18,224 associated features. Preliminary filtering entailed identifying patients with diabetes by using International Classification of Diseases (ICD) codes. The preliminary cohort was further filtered by requiring a diagnosis of non-insulin-dependent diabetes indicated by ICD codes as shown in Figure 2 (*n* = 24,104 T2D patients). The following comorbidities were identified for the UKBB T2D patients by using ICD codes: obesity, angina, chronic ischemic heart disease (HD), pulmonary HD, ischemic stroke, atherosclerosis, vision problems, Alzheimer’s disease, metabolic disorders, mild cognitive impairment, other types of HD, and renal failure. Cancer history, heart attack history, hypertension status, sex assigned at birth, ethnic background, smoking status, alcohol intake frequency, overall health rating, use of medication for hypertension and hypercholesterolemia, and lab test results were noted in the UKBB, and were extracted for the T2D patients filtered into the UKBB dataset. Age was calculated as the age of the individual on the date of their blood test that was used to diagnose their T2D, based on their date of birth.

#### 2.2.2. Data Processing and Feature Selection for the AoU Dataset

The AoU dataset was downloaded with data on 409,420 individuals. A T2D cohort (*n* = 29,480) was created in the AoU NIH Cohort Builder by including participants with the ICD code for diabetes mellitus (Figure 2). The data fields present in the UKBB dataset were identified for the AoU dataset, and features of interest were extracted from the NIH server.

#### 2.2.3. Data Harmonization to Create the Multinational Combined Dataset

As the UKBB and AoU datasets utilized differing measurement units for lab sample data, feature processing was performed to convert and match the measurement units for both datasets to those utilized by AoU. Further processing was performed on demographic survey questions to simplify or harmonize the data (as appropriate for the data type) in order to combine the UKBB and AoU datasets to create the multinational Combined dataset (Supplemental Figure S1), which allowed for direct comparisons of modeling inputs for the UKBB MLA, AoU MLA, and Combined MLA. Textural information was converted into numerical values, which involved encoding categorical features into binary values by one-hot encoding (Supplemental information), to prevent the MLAs from incorrectly interpreting the categories as having a meaningful order. 

Some features, such as sex assigned at birth, age, and comorbidity status, were used “as-is”, i.e., the input features were not combined or compared outside the context of the MLA. Features with more than 50% missingness were excluded. The MLA implicitly combined and compared thresholds within the decision trees (as discussed in Section 2.3 Methods: Model Training) to generate the final output. The MLA automatically learned these thresholds during the training process and no arbitrary configuration was performed.

After the input features were selected (Supplemental Figure S2) and harmonized, they were categorized as numerical or categorical. Numerical values were first standardized to bring all numerical features to a consistent scale (in this case, decimal values between 0 and 1). For a given feature, this standardization process, using the StandardScaler technique in Scikit-learn, removes the mean and scales values to the unit variance; this is equivalent to calculating the z-score for each value of the feature. Overall, this process gives all features a relatively standard normalized data distribution. Missing numerical values were filled using distance-based KNN imputation [53], separately for numerical and categorical features. Rather than imputing all missing values with the mean or median of all non-missing values of a given feature, KNN imputation finds the k nearest neighbors (via Euclidean distance or some other appropriate distance metric, calculated using all numerical or all categorical features) that lack missing data and then takes the mean or median of the feature of interest from those k nearest neighbors. In this case, KNNImputer within Scikit-learn was used for imputation, with the two nearest neighboring samples uniformly weighted and Euclidean distances calculated. Imputed categorical variables were rounded to the nearest integer. Subsequently, numerical and categorical features were all recombined into one dataset. Each dataset (UKBB, AoU, and Combined) was subsequently split into an 80–20 train-test set (as discussed in 2.1 Methods: Dataset). A forward feature selection process was employed to identify features that could enhance prediction performance. The removal of highly correlated features is important to reduce model training time and complexity through dimensional reduction, and retaining highly correlated features does not benefit model performance. Therefore, to reduce the number of highly correlated features, one feature from each pair with a Pearson correlation coefficient greater than 0.85 was removed. The feature importance was then evaluated for 5 to 50 features during each iteration of selection. For a specific iteration of feature selection, the single feature AUROC was first calculated for every feature. Then, the pairwise AUROC was computed for each combination of features. The features with the highest single feature and pairwise AUROCs were selected, given their high forecasted impact on classification performance (AUROC, NPV, and sensitivity). This process was repeated until a maximum of 50 features were chosen for each iteration, with fewer features selected if they did not contribute significantly to the prediction. The significance threshold was determined beforehand through parameter tuning. The features used in the forward feature selection are presented in the Supplemental information (Supplemental Figure S2). To address the class imbalance, with just under 8% of individuals in the Combined dataset having HT, the Synthetic Minority Over-sampling Technique (SMOTE) was applied [54]. Following these preprocessing steps, each MLA model was individually trained (as discussed in Section 2.3 Methods: Model Training).

### 2.3. Model Training

#### 2.3.1. Algorithm Selection

In order to select a classification approach, we compared the performances of several different ML models from Scikit-learn for the task of predicting HT risk in T2D patients: a random forest classifier, a KNN classifier, and a multilayer perceptron classifier (MLPClassifier). For this comparison, we used default parameters, except for the MLPClassifier, which was used with and without grid searching. For the random forest classifier, the default parameters include 100 trees comprising the forest, Gini impurity to measure the quality of each split, a minimum of two samples required to split an internal node, and the consideration of, at most, the square root of the total number of features when looking for the best split. For the KNN classifier, the default parameters include use of the five nearest neighbors in each query, uniform weighting of all points in each neighborhood, automated determination of which algorithm to use in computing the nearest neighbors, and use of the Euclidean distance. For the MLPClassifier, the default parameters include the use of a single hidden layer with 100 hidden units which utilizes a rectified linear unit function for the hidden layer, the use of the “adam” stochastic gradient-based optimizer for weight optimization, an alpha value (L2 regularization strength) of 0.0001, and a constant learning rate of 0.001. The MLPClassifier grid search covered the use of one or two hidden layers of sizes between 100 and 1000 hidden units, four different activation functions (no-op, logistic sigmoid, hyperbolic tan, and rectified linear unit), alpha values ranging from 1 × 10^−5^ to 0.1, and either a constant or an adaptive learning rate schedule, with the initial learning rate ranging from 1 × 10^−4^ to 0.1. We evaluated the model performance on the corresponding test set (i.e., *n* = 4821 for UKBB; *n* = 5896 for AoU; *n* = 10,717 for Combined), and the random forest algorithm was chosen as it had the best balance between performance across the three MLAs (UKBB, AoU, and Combined) and interpretability (via feature importance).

#### 2.3.2. UKBB MLA Training

To classify individuals as HT vs. non-HT within the UKBB dataset, we employed a random forest classifier algorithm on the corresponding training set (*n* = 19,283; Table 1, Figure 2). This algorithm utilized a subset of the input features to construct decision trees, where each tree employed a series of rules to assign a class (HT class or non-HT class) for each patient based on their feature values. This process was repeated to create an ensemble of decision trees, which collectively determined the final output of the model. The approach of utilizing multiple trees from a subset of input data allowed the model to perform classifications that accounted for the heterogeneity of feature values among HT patients. The random forest approach performed well due to its relative robustness in the presence of potential outliers, lack of assumptions by the model about the underlying data distribution, ability to handle collinearity between features, elimination of the need for rigorous feature selection prior to model training, and balance between interpretability and ability to handle nonlinear dependencies [55,56]. All of those benefits were important in the final decision to use a random forest classifier, as most other options for modeling approaches reduce interpretability, add unnecessary complexity, or are too basic for this particular classification task. For consistency, a similar hyperparameter optimization approach was implemented for the UKBB MLA as for the AoU MLA and Combined MLA, and this optimization process and choice of parameters are described in more detail below for the training of the Combined MLA. Subsequent to training, the UKBB MLA was evaluated on the corresponding test set (*n* = 4821).

#### 2.3.3. AoU MLA Training

The AoU MLA was trained similarly to the UKBB MLA by using a random forest classifier algorithm on the corresponding training set (*n* = 23,584; Table 1, Figure 2). As with the UKBB MLA, the optimization process and choice of model parameters is described in detail in the subsequent section about the training of the Combined MLA. After training, the AoU MLA was evaluated on the corresponding test set (*n* = 5896).

#### 2.3.4. Combined MLA Training

The Combined MLA was trained similarly to the UKBB MLA and the AoU MLA by using a random forest classifier algorithm on the corresponding training set (*n* = 42,867; Table 1, Figure 2). Before training the Combined MLA, hyperparameter optimization was performed via the cross-validation method of hyperparameter tuning by using the corresponding training set (*n* = 42,867). Cross-validation is a resampling method that uses different portions of the data (i.e., data subsets) to test and validate a model on different iterations. In this case, the 42,867 individuals in the Combined training set were divided into five random and approximately equal subsets, after which a random forest model was trained on four of them and validated on the remaining one. This was repeated using each of the five subsets as a validation set, where the subsets were not reshuffled between folds. The method of cross-validation allowed for the building of a model that was more robust to variability in the data, i.e., a more generalizable model. Hyperparameters were optimized using a grid search method from Scikit-learn, which takes each possible combination of hyperparameters and runs each combination through the cross-validation process [57]. The three main hyperparameters tuned using the grid search method were the number of decision trees, the maximum tree depth, and the minimum number of samples to split an internal node. The “number of decision trees” hyperparameter determines the number of trees that are built before averaging their predictions. More trees would improve performance, but also increase the risk of model overfitting and increase the overall training time. Thus, the search grid for the number of decision trees was kept between 100 and 500. The “maximum tree depth” hyperparameter determines the complexity of the weak learners—it limits the depth of the contributing decision trees, thereby controlling the number of features that are part of the classification of each weak learner. A relatively low range of values between 2 and 4 was selected for tuning maximum tree depth to develop a more conservative model, thus limiting weak learners that overfit to specific feature values of the training dataset. The “minimum number of samples to split an internal node” hyperparameter specifies the minimum number of samples that must be at an internal node to allow for a split at that node within a given decision tree. Values between 3 and 10 were selected for tuning the minimum number of samples to split an internal node. These values were chosen to avoid overfitting which may occur by splitting an internal node after very few samples were at the node, resulting in splits on extreme or rare values for an input feature. The final set of optimal hyperparameter values were as follows: number of decision trees = 500, maximum tree depth = 4, and minimum number of samples to split an internal node = 3. In hyperparameter optimization, three performance metrics were used in scoring—AUROC, NPV, and sensitivity—due to their applicability in healthcare screening, and the best combination of the hyperparameters investigated was selected for maximizing the average of those three metrics. Once the optimal hyperparameters were obtained, the Combined MLA was trained using the corresponding training set (*n* = 42,867), after which it was then evaluated on the corresponding test set (*n* = 10,717).

### 2.4. Model Performance Evaluation

After each prediction algorithm was trained, we tested the trained MLA on the corresponding test set (*n* = 10,717 for the Combined MLA; *n* = 4821 for the UKBB MLA; *n* = 5896 for the AoU MLA; Supplemental Table S1). The predictive performance of the trained MLA on its corresponding test set was evaluated using several standard performance measures [58,59,60]: AUROC (the probability that the classifier algorithm can distinguish between positive and negative examples), sensitivity (true positive rate/TPR or the percentage of actual positives that the model correctly identifies), specificity (true negative rate or ability of a model to correctly identify negative cases), positive predictive value (PPV, the proportion of true positives among all positive predictions), and NPV (the proportion of true negatives among all negative predictions).

The importance of each input feature (Supplemental Figure S2) to model performance was evaluated using SHapley Additive exPlanations (SHAP) values [54,60]. SHAP feature evaluation allows for an understanding of the most predictive features, where the SHAP values indicate the magnitude of a feature’s impact on the model’s performance. For MLAs, the SHAP values of all input features sum up to the difference between the baseline (expected) output of the MLA and the current (actual) predictions, with each individual SHAP value representing a single feature’s contribution to that deviation from the expected output. Each input feature is handled independently in determining its effects on the MLA’s predictions, thereby allowing for the investigation of each input feature’s importance independent of the effects of any other features.

### 2.5. Statistical Analysis

The confidence intervals (CIs) for the AUROCs were calculated using a bootstrapping method, where a subset of patients from the corresponding test set were randomly sampled and the AUROC was calculated using the data from those patients. This step was repeated 1000 times with replacement. From these 1000 bootstrapped AUROC values, the middle 95% range was selected to be the 95% CI for the AUROC. As the sample size of the test set for each of the three MLAs was greater than 30, the CIs for the other metrics (sensitivity, specificity, PPV, and NPV) were calculated using normal approximation.

### 2.6. System Requirements

In this study, we utilized a 2021 MacBook Pro equipped with 16 GB of memory and an Apple M1 Pro chip for training, testing, and performance evaluation. Training was completed in less than three seconds for each model at each time window. Since computer capacity is not a limiting factor for the ML analysis of tabular data, most modern Mac, Windows, and Linux computers with at least 8 GB of memory can easily handle the training of any of the models (random forest, KNN, and MLPClassifier) we describe here.

All data processing, training, testing, and evaluations were performed in Python. Only common scientific packages were used: pandas 2.1.2 for data input and manipulation and Scikit-learn 1.3.2 for ML using the MLA (random forest) and comparison models (KNN and MLPClassifier) [56,57,61,62]. NumPy 1.26.1 was employed for working with arrays, while Matplotlib 3.8.1 facilitated the creation of visualizations.

## 3. Results

We compared the performance of the different models (random forest, KNN, and MLPClassifier), and determined that the random forest model had the best overall performance across the three datasets (UKBB, AoU, and Combined; Supplemental Table S2). An MLP classifier is a feedforward artificial neural network model that maps the input dataset to a set of outputs using a network consisting of a series of dense hidden layers that extract information from the data, followed by a final “softmax” layer scaling outputs into a prediction (binary, in the case of our MLAs) [63]. MLP classifiers, although a powerful classification approach, have minimal or zero explainability—it is difficult or impossible to elucidate how such models make their classifications. A KNN classifier is a non-parametric, supervised lazy-learning classifier, where the model is not constructed until the prediction time and therefore can incur a substantial computational cost at the time of inference, the prediction requires having the full training set available throughout the model’s lifetime, and the algorithm is relatively sensitive to noise and local patterns [62]. By contrast, the random forest classifier is an eager-learning algorithm that constructs a generalized predictive model based on the training dataset and does not require access to the training set to deliver a prediction once trained [56]. Given these benefits of the random forest classifier over the KNN classifier and the MLPClassifier, and that the overall performance balance was the best for the random forest model (Supplemental Table S2), this algorithm was chosen to develop the three MLAs for our study (i.e., UKBB MLA, AoU MLA, and Combined MLA). 

The performance metrics of each of the three MLAs for differentiating between HT and non-HT for individuals with T2D are shown in Table 2. The MLAs are optimized to be used as screening tools for identifying individuals with T2D who have an elevated risk of developing HT. To this end, the metrics in Table 2 were calculated at the operating point at which the sensitivity and NPV were maximized for the specific MLA [64]. Maximizing sensitivity in a screening test ensures that few individuals with the target condition are missed (low false negatives), allowing for confidence in “ruling out” the condition of those who test negative which results in a high NPV [64]. The Combined MLA outperformed the AoU, MLA, and UKBB MLAs in predicting HT risk after a T2D diagnosis, with a higher AUROC value. By contrast to the other performance metrics, the AUROC has the advantage of being independent of a chosen classification threshold (i.e., a chosen operating point) [65]. Therefore, although selecting an operating point gives a snapshot set of sensitivity, specificity, PPV, and NPV values to assess MLA performance, the AUROC is a more robust assessment metric, allowing for the overall analysis of a model’s inherent validity and enabling a broader comparison between different MLAs [58,65]. Notably, the Combined MLA displayed a very high NPV of 0.987 (95% CI: 0.983–0.991) in addition to the highest AUROC of 0.762 (95% CI: 0.747–0.778), which indicates the suitability of the Combined MLA for use as a screening CDS tool for the reliable identification of T2D patients with a low risk of developing HT.

**Figure 3:**
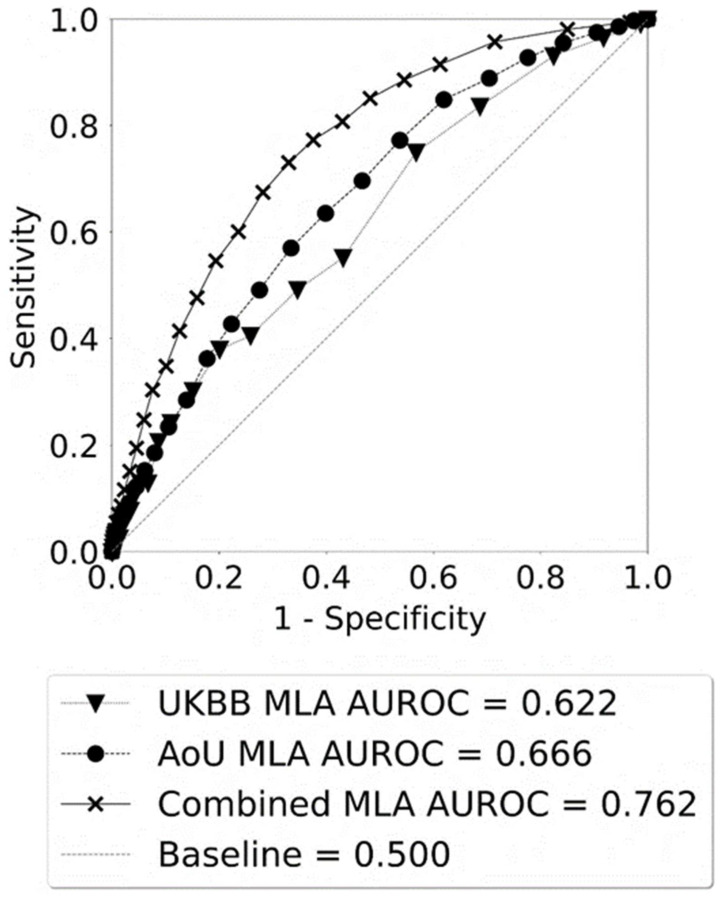
The receiving operating characteristic (ROC) curves for hypothyroidism (HT) vs. non-HT as given by the three machine learning algorithms (MLAs), when each prediction algorithm was tested on the corresponding test set.

Figure 3 shows the receiver operating characteristic (ROC) curves for predicting HT vs. non-HT for each of the three MLAs. The ROC curves plot the TPR against the false positive rate (FPR) and indicate how well a binary classifier can distinguish between two classes at different thresholds. The baseline curve represents a model that is similar to a random coin flip (0.5, dashed line). For predicting HT in T2D individuals, the Combined MLA performed significantly better than the baseline, displaying the highest AUROC. In contrast, the UKBB MLA and the AoU MLA had significantly lower AUROC values of 0.622 (95% CI: 0.573–0.671) and 0.666 (95% CI: 0.646–0.687), respectively. This suggests that employing a larger dataset consisting of intrinsically broader information and patient demographics to develop an MLA may result in a more robust MLA. Unlike the AoU, MLA, and Combined MLA ROC curves, the UKBB ROC curve has a visible deviation (“lump”) in its curvature between 0.3 and 0.4 on the *x*-axis (1—specificity) which is common in models derived from datasets with a low proportional representation of the positive class, a value which is particularly low in the UKBB dataset (1.9% HT in the UKBB dataset versus 12.7% HT in the AoU dataset). This low proportional representation results in more jagged “steps” in the curve, since moving between any two points involves a relatively small number of positive cases, and noisiness in the data of those cases may contribute to an outsized visual effect on the curve.

**Figure 4:**
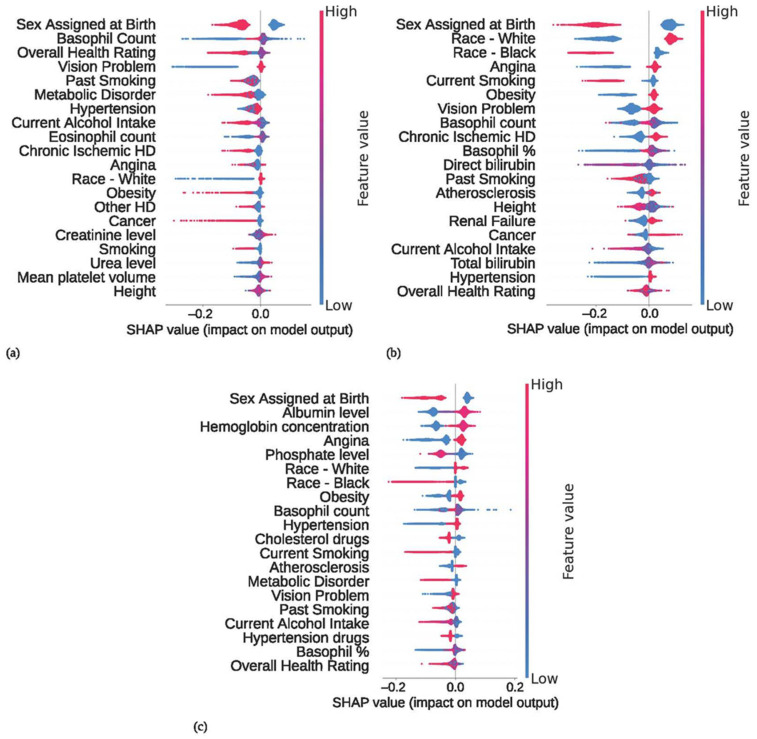
SHapley Additive exPlanations (SHAP) beeswarm summary plots visualizing feature importance for the (**a**) UKBB MLA, (**b**) AoU MLA, and (**c**) Combined MLA. HD = heart disease.

The top input features that influenced the performance of each MLA were evaluated using SHAP values, which helped us to interpret the importance of these features to the output of each MLA. The SHAP values ranked the input features, highlighting their significance in the model and their contribution to the MLA’s predictive performance. The higher the absolute value of SHAP values for a given feature relative to other features, the more important (substantial) the contribution of that feature is to the MLA’s predictive performance. A feature with a positive SHAP value leads the MLA to predict the positive class (in this case, HT), with a feature having a more positive SHAP value contributing more substantially to this positive class prediction. A feature with a negative SHAP value leads the MLA to predict the negative class (in this case, non-HT), with a feature having a more negative SHAP value contributing more substantially to this negative class prediction. The top three predictive features were sex assigned at birth, basophil count, and overall health rating for the UKBB MLA; and sex assigned at birth, White racial identity, and Black racial identity for the AoU MLA. For the Combined MLA, the top three features were sex assigned at birth, blood albumin level, and hemoglobin concentration. The top predictive feature for all three models was sex assigned at birth, which correlates with the existing literature indicating that females are at a higher risk of developing HT comorbid with T2D; research by Song et al. demonstrated an odds ratio of 2.02 associated with females vs. males [66]. To assess the contribution of sex assigned at birth to the MLA predictions, the ROC curves for the univariate model (UVM) solely utilizing sex assigned at birth as the predictor were compared to the corresponding MLA ROC curves (Supplemental Figure S3). When compared to the multivariate UKBB MLA, the UKBB UVM showed no significant difference in AUROC value. The AoU UVM and the Combined UVM displayed significantly lower AUROC values when compared to the corresponding multivariate MLAs (i.e., 0.571 vs. 0.666 for AoU, and 0.613 vs. 0.762 for Combined, respectively). Additionally, other performance metrics such as NPV and sensitivity were significantly lower for both the AoU UVM and Combined UVM compared to their corresponding multi-variable MLAs (Supplemental Table S3). These results indicate that the distinction lies in the remaining features, highlighting specific differences between the datasets with respect to the features that enhance the predictive power of each model. The feature importance, overall feature ranking, and how each feature influences the output predictions are depicted in the corresponding SHAP beeswarm summary plots for each MLA (Figure 4), which provide a visual interpretation of the contributions of the top input features in classifying patients as HT vs. non-HT. Each point on a SHAP summary plot represents the SHAP value of a particular feature for a single T2D patient, a value that quantitatively measures the importance of a feature for a specific prediction. In total, SHAP summary plots showcase the relationships between the inputs and the output predictions across all data vectors that are fed to the model. Smaller SHAP values (more negative) indicate a correlation with the negative class (non-HT) while larger values (more positive) indicate a correlation with the positive class (HT). Generally, a SHAP value close to 0 for a feature indicates that, for a single individual, the value of that feature is less predictive. SHAP further provides insights into how the specific values of features correlate with the model’s predictions through the use of color to indicate high (red) or low (blue) values for the normalized values of a particular feature, with shades of purple indicating values in the middle for continuous or ordinal inputs. For the data displayed in Figure 4, the maximum possible absolute value for a SHAP value is 1, since individuals are labeled as 0 (non-HT cohort) or 1 (HT cohort). 

## 4. Discussion

This study aimed to provide the proof-of-concept for a CDS tool that may enhance the ability of clinicians to determine if a patient is at risk of developing HT comorbid with T2D using readily available patient data and in settings where clinicians do not have specialized training in endocrine disorders or on-site access to labs. The current SOC for HT screening in T2D individuals in the US calls for TSH testing at the time of diagnosis and every five years thereafter [10,67], in part, to minimize patient costs [10,67]. However, given the risk for severe diabetic complications in patients with comorbid HT and T2D [22], more frequent screening may provide benefits in terms of care coordination. This work fills a significant gap that can address limitations that exist with current SOC screening recommendations, as well as provide the basis for future validation and implementation of an HT-risk screening method for individuals with T2D. Further, we present an MLA with strong clinical utility, as it is a software-only tool that can be implemented as a cloud-based or app-based solution to allow any clinician, regardless of medical specialty or expertise, to utilize this screening approach. The automation of screening and patient care processes is already enabling healthcare professionals to focus on more complex patient needs, which can improve diagnostic accuracy and alleviate the burden on the healthcare system [68].

The Combined MLA outperformed the UKBB MLA and the AoU MLA in terms of performance metrics, achieving a significantly higher AUROC while maintaining a high NPV and high sensitivity. The higher AUROC indicates that the Combined MLA can provide tangible gains in identifying T2D individuals who may be at risk for developing HT. The high NPV and sensitivity together indicate that there is only a small percentage of false negatives among all individuals with negative test results and among all true positive individuals, respectively, which are desirable characteristics for a screening tool. Traditionally, testing for HT requires measuring TSH levels in blood, which is invasive as the test requires a blood sample [69]. Additionally, TSH testing is not routinely performed like a complete blood count or a complete metabolic panel, for example. A CDS tool based on the Combined MLA can supplement the current SOC to provide risk assessments within diverse clinical settings at more regular intervals and without requiring additional patient expenditures and/or the time and travel burdens posed by the need to visit clinical laboratories. This, in turn, could help reduce overall healthcare costs with early intervention, as T2D and HT comorbidity increase the risk of debilitating, expensive, and often irreversible diabetic complications [15,18,20,22].

A strong asset of the Combined MLA as a CDS tool is its high NPV (0.987). Screening tests that yield high NPVs can be of value in clinical practice to confidently exclude patients who are not at risk of developing a certain condition [64]. Low-precision, high-recall (relatively high NPV and low PPV) screening methods focus on maximizing the detection of a medical condition by prioritizing sensitivity over specificity, particularly for low-prevalence conditions. Patients who receive a negative result can trust its veracity, while some of the patients who are recalled for further investigation based on a potentially positive result will also receive a negative result, allowing for the identification of the true positive cases during further investigations. For example, a mammogram to rapidly screen patients for the possible presence of breast cancer boasts a very high NPV but a relatively low PPV [70]. As another example, in the case of HIV, with a regionally variable prevalence rate that ranges from 0.1% to over 20%, low-cost high NPV tests (>99%) such as the First Response HIV-1-2 antigen test allow for mass screening [71,72]. In these contexts, a high number of true negatives are correctly identified, leading to reduced unnecessary additional testing for a large portion of the T2D population. 

Aligning with this concept of NPV being a valuable method to rule out conditions in patients, the NPV achieved by the Combined MLA indicates its potential value in identifying individuals who are not at risk for developing HT. With HT prevalence being relatively low among adults with T2D, only at 12% [13,14], a low-precision, high-recall CDS tool for HT risk screening in T2D patients would allow clinicians to focus resources on the smaller portion of T2D patients with a higher HT risk. This would allow T2D patients with a lower HT risk to avoid unnecessary lab testing. Out of the 10,717 individuals in its hold-out test set, the Combined MLA correctly identified 7504 true negatives (accounting for 70.0% of the patients), with only 357 false negatives (Supplemental Figure S4). Given the relatively low HT prevalence among T2D patients (below 20%), the MLA’s high NPV can effectively indicate a low HT risk in a substantial proportion of T2D patients (>80% of T2D patients never manifest HT). The operating point was chosen to prioritize true negatives and this is reflected in our findings that the majority of misclassifications in the hold-out test dataset were false positives. This is crucial for a medical-use low-precision, high-recall CDS tool, as false negatives can lead to delayed treatment and poorer patient outcomes; a large number of false positives (high-recall) decreases the possibility of missing actual positive cases, by ”recalling” patients for secondary testing, where the benefits of testing the false positives outweigh missing the true positives [73,74]. To our knowledge, there has not been any other approach that attempted to predict conditions comorbid with T2D; therefore, the Combined MLA could potentially fill a gap in screening protocols and for validating tools that address comorbidities with T2D for a wide population of T2D patients compared to the SOC.

The SHAP analysis identified several important features that contributed significantly to the MLA predictions. The top features across all MLA models were sex assigned at birth, racial identity, and several blood test results, such as basophil count values. In terms of sex assigned at birth, there is documented evidence that females in the general population (non-T2D specific) have a higher HT risk when compared to males [75], and this risk increases even more in females with T2D in comparison to males with T2D [76]. This elevated risk for the T2D female population is reflected by sex assigned at birth emerging as the most important input feature to our MLAs across all datasets in predicting HT risk in T2D patients. This is further highlighted by our findings that a sex-only predictor UVM model displayed a substantially similar AUROC value to the UKBB MLA, indicating that the contribution of the other input features to the UKBB MLA predictions did not add substantial predictive value. The AUROC values for the sex-only AoU UVM and the Combined UVM compared to the respective MLAs were significantly lower, indicating that for the AoU MLA and Combined MLA, the input features other than sex assigned at birth had a meaningful contribution to the predictions. The AoU dataset had female individuals among those without HT (55.6% female) overrepresented compared to the US population with T2D (45.2% female) [2], while the UKBB dataset (36.0% female) had female individuals underrepresented compared to the UK population with T2D (44.2% female) [77]; although the split of sex assigned at birth is similar for HT patients in both datasets, the underrepresentation of females in the UKBB may have reduced MLA performance fairly substantially. As females with T2D are much more likely than males with T2D to develop HT, it is especially important for females to be adequately represented to prevent dataset biases that may perpetuate into the model. In all three MLAs, sex assigned at birth was the most important feature for the classification of HT versus non-HT individuals, but it was more important in the AoU MLA than in the UKBB MLA or the Combined MLA.

Racial identity also played a notable role in predicting HT risk in T2D patients in our MLA, which correlates with documented varying levels of risk for T2D and T2D-related complications (including HT) depending on an individual’s ethnic identity [78]. Specifically, HT risk is lower in the Black population than in the White population [79] and this is further reflected in the racial identity-based feature importance shown in the SHAP plots of Figure 4. As previously discussed, input features other than sex assigned at birth did not contribute meaningfully to the UKBB MLA predictions, and this may be in part due to the underrepresentation of racial minorities in the UKBB. Thus, we discuss below the contribution of input features other than sex assigned at birth only for the predictive power of the AoU MLA and the Combined MLA. Racial identity significantly enhances the predictive power of both the AoU MLA and the Combined MLA, with White patients being associated with a higher HT risk in Figure 4a and Figure 4c, respectively; this is likely at least in part due to the more diverse demographics of the AoU and Combined datasets. Compared to the US general population [2], in the AoU dataset, White individuals (60.5% versus 44.7%, respectively) and Asian individuals (6.1% versus 2.0%, respectively) with T2D were underrepresented, while Black individuals (13.6% versus 28.8%, respectively) were overrepresented, which resulted in the AoU dataset providing an overall better balance of racial identities for the purposes of MLA training compared to the UKBB dataset. For the UKBB dataset, the dataset population (87.6% White, 3.2% Black, and 5.3% Asian) was significantly less diverse than the UK population with T2D (24.0% of minority ethnic origin) [77], and this lack of diversity may have contributed to the observed performance of the UKBB MLA.

Basophil count ranked high in feature importance (Figure 4), and basophilic disorders with an increased circulating basophil count have been previously reported to be associated with HT [80,81]. Basophil count enhanced the predictive power of the AoU MLA and to a lesser degree, the Combined MLA. Smoking was also found to be an important input feature associated with a decreased HT risk (Figure 4), which aligns with research indicating that tobacco smoking is associated with a reduced risk for HT, possibly due to the stimulatory effect of nicotine on the metabolism [82]. Current smoking status had a significant contribution to the AoU and Combined MLA predictions.

Several possible factors may have contributed to the better performance of the Combined MLA compared to both the AoU and UKBB MLAs. First and foremost, a larger training dataset often leads to better MLA generalization as long as the additional data are of similar quality, which is especially necessary when dealing with relatively rare events, such as HT occurring in patients with T2D. This is in part due to a larger training dataset providing a relatively larger number of both common and uncommon types of examples for the modeling approach to learn from [83,84]; this is particularly important for individuals with relatively rare conditions (e.g., T2D patients with HT), for which even a small number of additional training examples may substantially benefit predictive performance. Further, more data exposure enables the identification of trends and important features which may only have a handful of relevant examples in a smaller dataset but can surface as more prominent patterns in a larger dataset. Interestingly, albumin, hemoglobin, and phosphate levels emerge as the second, third, and fifth most important features contributing to the predictive power of the Combined MLA, despite none of those three features ranking among the top 20 most important for either the AoU ML or the UKBB MLA. Disruptions in hemoglobin and albumin homeostasis are known risk factors for HT, where anemia and high levels of glycated blood albumin concentrations correlate with an increased risk of HT [85,86,87]. This highlights that expanding the dataset size and diversity may uncover crucial predictive features that could remain hidden when using an MLA developed based on smaller datasets.

A larger, more diverse training set also reduces overfitting, thus making MLAs more robust when they are tested. Additionally, the component datasets of the Combined dataset are sampled from different/multinational populations, with the AoU dataset originating from the US and the UKBB dataset from the UK. Each component dataset contributes relatively elevated proportions of certain demographic groups to the Combined dataset (e.g., a higher proportion of Ashkenazi Jewish and Black individuals from the US, and more South Asian and Welsh individuals from the UK), thus providing increased demographic diversity to the Combined dataset by comparison with each of the component datasets.

Research by the US Government Accountability Office indicates that AI-based tools in healthcare can reduce the burden on the healthcare system, cut costs, better predict health conditions, and improve treatment plans [88]. Research on MLA-based predictive tools in the context of T2D and HT does not exist, to our knowledge. However, research on predictive ML for use with T2D and HT, separately, may provide a preliminary basis for the development and validation of tools that are specific to these two disorders in the context of co-occurrence. For example, several studies have employed novel approaches to overcome classification challenges that exist in research on predictive MLAs for diabetes by using big data for model development and validation. These challenges include the need for invasive input data to generate predictions [69] and lack of output interpretability [89]. Some of these approaches have included ensemble learning [43] and the implementation of Local Interpretable Model-Agnostic Explanations and prediction results within smartphone applications for easy-to-interpret results [89]. In regard to HT prediction using ML, studies have worked to overcome diagnostic challenges that pertain to equivocal symptoms [43], and challenges related to the data itself, such as class imbalances [90]. This was achieved, respectively, by elucidating important symptoms that contribute to HT and non-HT classification [43] and generating artificial data to include model development and testing using a Conditional Tabular Generative Adversarial Network [90]. Similar to our use of distinct datasets to test how diversified data impact model performance, Hu et al. used patient data from four clinical sites to examine the generalizability of an ML to predict thyroid disease; the model performed well, with an AUROC of 90.9%, and the external validity was confirmed by the lack of bias within the different sites [91]. This study, however, focused on a population within a single country largely represented by a homogenous population, making it difficult to ascertain the global generalizability of this technique and the model itself. Though these studies represent important contributions to understanding and classifying T2D and HT separately, the fact remains that no studies exist that examine the ML-based predictability of these two disorders together.

## 5. Current Limitations

This proof-of-concept study demonstrates that an MLA-based CDS tool can provide automated screening of individuals with T2D who may be at risk of developing HT; however, our study has several limitations. The datasets used for this study are composed of retrospective patient data, and to determine the clinical effectiveness of the Combined MLA for HT risk prediction in T2D patients, the MLA needs to be validated using prospective patient data. While the AoU and UKBB datasets are invaluable in research for comorbid prediction analysis, the data were collected from US and UK clinical sites, and thus do not account for regional and population-specific variations, particularly for variability in patient data, clinical testing guidelines, and treatment protocols that may lead to differences in HT risks [21]. Specifically, in the UKBB, there was a markedly lower number of T2D patients who were diagnosed with HT compared to the AoU database. This could be the result of different screening practices in the UK, where TSH screening is indicated only at the time of T2D diagnosis, with no further screening, which may lead to potential HT underdiagnosis [21]. Additionally, there were some differences regarding the racial demographics of the AoU dataset and the general population of the US due to the AoU research program’s efforts to increase the number of participants who were historically underrepresented in biomedical research [52]. These differences in demographics can restrict the generalizability of the research findings.

## 6. Conclusions

Comorbid T2D and HT are an under-recognized and potentially underestimated healthcare concern, which can result in a loss in quality of life for the patient and excess healthcare expenditures. Current guidelines for HT testing in T2D individuals likely lead to HT underdiagnosis. The Combined MLA, trained on data harmonized from two large multinational datasets, had significant performance gains compared to the AoU and UKBB MLAs, particularly in terms of the AUROC (relative to both other MLAs) and sensitivity (relative to the UKBB MLA). This predictive model also reflects clinically relevant risk factors for HT in patients with T2D, with several of those clinical features not as readily identified by models trained on the smaller component datasets (the AoU and the UKBB datasets). Our Combined MLA could be employed as a low-precision, high-recall CDS tool with a high NPV that effectively screens out a relatively large volume of T2D patients with low HT risk. Future work to further validate this MLA will entail the prospective evaluation of diverse datasets to determine generalizability and determine the characteristics of patients that contribute to their HT risk profile to enhance the interpretability of the model output.

## Figures and Tables

**Table 1:** A demographics table for the training sets. Each integer value represents a count for a given column (e.g., HT subjects within the UKBB dataset), while each percentage represents the percent of all subjects in that column having a particular demographic feature (e.g., female sex assigned at birth).

Demographics (Training Set)		UKBB Dataset		AoU Dataset		Combined Dataset	
		HT (*n* = 380)	non-HT (*n* = 18,903)	HT (*n* = 2997)	non-HT (*n* = 20,587)	HT (*n* = 3320)	non-HT (*n* = 39,547)
Age (years)	22–40	0 (0.0%)	0 (0.0%)	377 (12.6%)	2810 (13.6%)	365 (11.0%)	2789 (7.1%)
	41–50	0 (0.0%)	125 (0.7%)	619 (20.7%)	4331 (21.0%)	580 (17.5%)	4451 (11.3%)
	51–60	39 (10.3%)	2291 (12.1%)	985 (32.9%)	6564 (31.9%)	1005 (30.3%)	8956 (22.6%)
	61–70	143 (37.6%)	6409 (33.9%)	752 (25.1%)	5020 (24.4%)	921 (27.7%)	11,473 (29.0%)
	71–80	198 (52.1%)	10,048 (53.2%)	238 (7.9%)	1635 (7.9%)	423 (12.7%)	11,631 (29.4%)
	>80	0 (0.0%)	30 (0.2%)	26 (0.9%)	227 (1.1%)	26 (0.8%)	247 (0.6%)
Sex assigned at birth	Female	213 (56.1%)	6724 (35.6%)	2060 (68.7%)	11,094 (53.9%)	2222 (66.9%)	17,804 (45.0%)
	Male	167 (43.9%)	12,179 (64.4%)	866 (28.9%)	9058 (44.0%)	1029 (31.0%)	21,297 (53.9%)
	Unknown	0 (0.0%)	0 (0.0%)	71 (2.4%)	435 (2.1%)	69 (2.1%)	446 (1.1%)
Racial identity	White	333 (87.6%)	16,569 (87.7%)	1756 (58.6%)	8812 (42.8%)	2033 (61.2%)	25,315 (64.0%)
	Black	10 (2.6%)	624 (3.3%)	528 (17.6%)	6175 (30.0%)	519 (15.6%)	6890 (17.4%)
	Asian	25 (6.6%)	980 (5.2%)	66 (2.2%)	413 (2.0%)	96 (2.9%)	1398 (3.5%)
	More than one race	0 (0.0%)	78 (0.4%)	32 (1.1%)	251 (1.2%)	33 (1.0%)	325 (0.8%)
	Other	9 (2.4%)	285 (1.5%)	42 (1.4%)	369 (1.8%)	55 (1.7%)	676 (1.7%)
	Unknown	3 (0.8%)	367 (1.9%)	573 (19.1%)	4567 (22.2%)	584 (17.6%)	4943 (12.5%)
Substance use (Yes/No)	Current smoker	44 (11.6%)	2446 (12.9%)	312 (10.4%)	3191 (15.5%)	342 (10.3%)	5648 (14.3%)
	Unknown smoking status	0 (0.0%)	81 (0.4%)	1671 (55.8%)	11,372 (55.2%)	1635 (49.2%)	11,524 (29.1%)
	Ever smoked	176 (46.2%)	8798 (46.5%)	1332 (44.4%)	9254 (45.0%)	1480 (44.6%)	17,985 (45.5%)
	Unknown smoking history	0 (0.0%)	0 (0.0%)	72 (2.4%)	508 (2.5%)	79 (2.4%)	519 (1.3%)
	Currently frequently use alcohol	87 (22.9%)	5860 (31.0%)	159 (5.3%)	1268 (6.2%)	244 (7.3%)	7105 (18.0%)
	Unknown alcohol status	0 (0.0%)	100 (0.5%)	456 (15.2%)	3512 (17.1%)	452 (13.6%)	3668 (9.3%)
Medications	Cholesterol	259 (68.2%)	12,208 (64.6%)	847 (28.3%)	5470 (26.6%)	1121 (33.8%)	17,716 (44.8%)
	Hypertension	227 (59.7%)	11,449 (60.6%)	1062 (35.4%)	6415 (31.2%)	1281 (38.6%)	17,952 (45.4%)
Comorbidities	Obesity	67 (17.6%)	3211 (17.0%)	2108 (70.3%)	12,887 (62.6%)	2129 (64.1%)	16,048 (40.6%)
	Angina	84 (22.1%)	3738 (19.8%)	2112 (70.5%)	12,480 (60.6%)	2166 (65.2%)	16,261 (41.1%)
	Chronic ischemic HD	101 (26.6%)	5192 (27.5%)	1321 (44.1%)	6814 (33.1%)	1374 (41.4%)	12,058 (30.5%)
	Pulmonary HD	10 (2.6%)	647 (3.4%)	192 (6.4%)	765 (3.7%)	197 (5.9%)	1392 (3.5%)
	Atherosclerosis	4 (1.1%)	181 (1.0%)	1417 (47.3%)	7608 (37.0%)	1369 (41.2%)	7864 (19.9%)
	Vision problem	310 (81.6%)	14,847 (78.5%)	1405 (46.9%)	7889 (38.3%)	1658 (50.0%)	22,827 (57.7%)

AoU = All of Us, HD = heart disease, HT = hypothyroidism, UKBB = UK Biobank.

**Table 2:** The performance metrics of the three MLAs on the corresponding test sets. Each AUROC is presented as the mean of 1000 bootstrapped AUROC values along with the 95% CI (the middle 95% of the 1000 bootstrapped values). The 95% CIs for specificity, specificity, PPV, and NPV were calculated using normal approximation.

Performance Metrics	UKBB MLA	AoU MLA	Combined MLA
AUROC (95% CI)	0.622 (0.573–0.671)	0.666 (0.646–0.687)	0.762 (0.747–0.778)
Sensitivity (95% CI)	0.655 (0.577–0.733)	0.997 (0.995–1.000)	0.965 (0.955–0.976)
Specificity (95% CI)	0.489 (0.476–0.502)	0.035 (0.032–0.038)	0.238 (0.230–0.245)
PPV (95% CI)	0.031 (0.025–0.037)	0.126 (0.120–0.131)	0.103 (0.097–0.109)
NPV (95% CI)	0.983 (0.978–0.988)	0.989 (0.979–0.999)	0.987 (0.983–0.991)

AoU = All of Us, AUROC = area under the receiver operating characteristic, CI = confidence interval, MLA = machine learning algorithm, NPV = negative predictive value, PPV = positive predictive value, UKBB = UK Biobank.

## Data Availability

Both datasets (AoU and UKBB) used for this study are publicly available for researchers who can provide appropriate credentials and research interests.

## Supplementary Materials

The following supporting information can be downloaded at https://www.mdpi.com/article/10.3390/diagnostics14111152/s1; Supplementary materials accompany this manuscript and show additional demographic information, information pertaining to the process of data harmonization, MLA inputs, and additional performance metrics. Table S1: Hold-out test sets demographics table. Each integer value represents a count for a given column (e.g., HT subjects within the UKBB dataset), while each percentage represents the percent of all subjects in that column having a particular demographic feature (e.g., female sex assigned at birth). Table S2: Performance metrics of three candidate ML approaches on the test sets for each of the three datasets. Each AUROC is presented as the mean of 1000 bootstrapped AUROC values along with the 95% CI (the middle 95% of the 1000 bootstrapped values). The 95% CIs for specificity, specificity, PPV, and NPV were calculated using normal approximation. Table S3: Performance metrics of the three UVMs, using only sex assigned at birth as the model input to predict HT status in patients with T2D, trained and tested on the same training and test sets as the corresponding multi-variable MLAs. Each AUROC is presented as the mean of 1000 bootstrapped AUROC values along with the 95% CI (the middle 95% of the 1000 bootstrapped values). The 95% CIs for specificity, specificity, PPV, and NPV were calculated using normal approximation. Figure S1: Details on harmonization of the UK Biobank (UKBB) and All of Us (AoU) datasets. MENA = Middle East and North Africa; NHPI = Native Hawaiian and Pacific Islander; T2D = type 2 diabetes. Figure S2: Machine learning algorithm (MLA) inputs. Figure S3: The receiver operating characteristic (ROC) curves for HT vs. non-HT for each MLA and the corresponding univariate model (UVM, using only sex assigned at birth as a model input to predict HT in patients with T2D) for the (A) UKBB dataset, (B) AoU dataset, and (C) Combined dataset. Error bars represent 95% confidence intervals (CIs) for sensitivity at each operating point displayed. AoU = All of Us; HT = hypothyroidism; MLA = machine learning algorithm; UKBB = UK Biobank. Figure S4: Confusion matrix showcasing the ability of the Combined MLA to reliably identify a high volume of T2D patients as having a low HT risk. HT = hypothyroidism; MLA = machine learning algorithm; NPV = negative predictive value; PPV = positive predictive value; T2D = type 2 diabetes.
