# Supplementary material for: Machine Learning Approach with Harmonized Multinational Datasets for Enhanced Prediction of Hypothyroidism in Patients with Type 2 Diabetes
Source: Diagnostics (Basel). 2024 May 31;14(11):1152. doi: 10.3390/diagnostics14111152 (PMC11172278; doi:10.3390/diagnostics14111152)
Supplement: Supplementary file 1 [file diagnostics-14-01152-s001.zip › diagnostics-3018935-supplementary.pdf]

Supplemental Table S1: Hold-out test sets demographics table. Each integer value represents a count for a given column (e.g., HT subjects within the UKBB dataset), while each percentage represents the percent of all subjects in that column having a particular demographic feature (e.g., female sex assigned at birth).

Demographics (Testing set)		UKBB dataset		AoU dataset		Combined dataset	
		**HT (n=116)**	**non-HT (n=4705)**	**HT (n=721)**	**non-HT (n=5175)**	**HT (n=894)**	**non-HT (n=9823)**
Age (years)	22-40	0 (0.0%)	0 (0.0%)	97 (13.5%)	694 (13.4%)	109 (12.2%)	715 (7.3%)
	41-50	1 (0.9%)	29 (0.6%)	127 (17.6%)	1079 (20.9%)	167 (18.7%)	1113 (11.3%)
	51-60	9 (7.8%)	553 (11.8%)	237 (32.9%)	1703 (32.9%)	265 (29.6%)	2155 (21.9%)
	61-70	47 (40.5%)	1640 (34.9%)	198 (27.5%)	1244 (24.0%)	219 (24.5%)	2840 (28.9%)
	> 70	59 (50.1%)	2483 (52.8%)	62 (8.6%)	455 (8.8%)	134 (15.0%)	3000 (30.5%)
Sex assigned at birth	Female	75 (64.7%)	1686 (35.8%)	466 (64.6%)	2765 (53.4%)	592 (66.2%)	4465 (45.5%)
	Male	41 (35.3%)	3019 (64.2%)	230 (31.9%)	2294 (44.3%)	275 (30.8%)	5253 (53.5%)
	Unknown	0 (0.0%)	0 (0.0%)	25 (3.5%)	116 (2.2%)	27 (3.0%)	105 (1.1%)
Racial identity	White	96 (82.8%)	4109 (87.3%)	426 (59.1%)	2198 (42.5%)	578 (64.7%)	6373 (64.9%)
	Black	4 (3.4%)	136 (2.9%)	122 (16.9%)	1570 (30.3%)	145 (16.2%)	1615 (16.4%)
	Other	14 (12.0%)	349 (7.4%)	39 (5.4%)	232 (4.5%)	43 (4.8%)	558 (5.7%)
	Unknown	2 (1.7%)	111 (2.4%)	134 (18.9%)	1175 (22.7%)	128 (14.3%)	1277 (13.0%)
Substance use (Yes/No)	Current smoker	12 (10.3%)	612 (13.0%)	71 (9.8%)	791 (15.3%)	97 (10.9%)	1392 (14.2%)
	Unknown smoking status	1 (0.9%)	25 (0.5%)	402 (55.8%)	2867 (55.4%)	439 (49.1%)	2821 (28.7%)
	Ever smoked	52 (44.8%)	2169 (46.1%)	318 (44.1%)	2321 (44.9%)	398 (44.5%)	4557 (46.4%)
	Currently frequently use alcohol	24 (20.7%)	1389 (29.5%)	40 (5.5%)	350 (6.8%)	66 (7.4%)	1762 (17.9%)
	Unknown alcohol status	1 (0.9%)	26 (0.6%)	117 (16.2%)	868 (16.8%)	122 (13.6%)	838 (8.5%)
Medi-cations	Cholesterol	72 (62.1%)	3005 (63.9%)	242 (33.6%)	1362 (26.3%)	299 (33.4%)	4329 (44.1%)
	Hypertension	73 (62.9%)	2855 (60.7%)	275 (38.1%)	1600 (30.9%)	356 (39.8%)	4367 (44.5%)
Comorb-idities	Obesity	18 (15.5%)	768 (16.3%)	505 (70.0%)	3244 (62.7%)	569 (63.6%)	4062 (41.4%)
	Angina	28 (24.1%)	966 (20.5%)	522 (72.4%)	3067 (59.3%)	580 (64.9%)	3990 (40.6%)
	Chronic ischemic HD	36 (31.0%)	1311 (27.9%)	292 (40.5%)	1724 (33.3%)	376 (42.1%)	2983 (30.4%)
	Pulmonary HD	2 (1.7%)	151 (3.2%)	46 (6.4%)	179 (3.5%)	53 (5.9%)	350 (3.6%)
	Atherosclerosis	1 (0.9%)	45 (1.0%)	324 (44.9%)	1922 (37.1%)	377 (42.2%)	1892 (19.3%)
	Vision problem	87 (75.0%)	3730 (79.3%)	335 (46.5%)	1981 (38.3%)	479 (53.6%)	5620 (57.2%)

AoU = All of Us; HD = heart disease; HT = hypothyroidism; UKBB = UK Biobank.

Data harmonization to create the Combined dataset

The process of data harmonization was carried out between the two datasets (UKBB and AoU) as follows: for the variable "Current Tobacco Smoking," the UKBB dataset had options such as "no answer," "no," "only occasionally," and "yes on most or all days." The AoU dataset options for responses about tobacco use were "not at all," "some days," "every day," "prefer not to answer," and "skip." To harmonize these datasets, responses from AoU were changed to match the language used in the UKBB dataset (Supplemental Fig. 1). In terms of "Alcohol Intake Frequency," UKBB used the response options of "no answer," "never," "special occasions only," "1-3 times a month," "1-2 times a week," "3-4 times a week," and "daily or almost daily." AoU options were "never," "monthly or less," 2-4 per month;" "2-3 per month," "4 or more per week," "prefer not to answer," and "skip." To harmonize these responses, categories from both datasets were matched accordingly (Supplemental Fig. 1). For the variable "Overall health Rating," the UKBB dataset had options like "no answer," "don't know," "poor," "fair," "good," and "excellent." The AoU dataset had options such as "poor," "fair," "good," "very good," "excellent," and "skip." To harmonize these, identical answers between the datasets were matched, and the categories like "good" and "very good" from AoU were combined into "good". A similar process of harmonization was followed for other variables such as hearing difficulty, ethnic background, and sex assigned at birth. Responses such as "no answer," "skip," and "don't know" were marked as N/A and thus, were not included in the harmonization list.

Textural information was then converted into numerical values, which involved encoding categorical features into binary values. The feature "sex" was encoded from "male"/"female" to "0"/"1." Similarly, binary values such as comorbidities were encoded from "yes"/"no" to "1"/"0." For unordered categories such as "racial identity," the labels such as "White," "Black," "Asian," etc., were converted into individual binary columns for each label (e.g., "White (0/1)," "Black (0/1)," "Asian (0/1)," etc.). This process, known as one-hot encoding, prevents the machine learning algorithm (MLA) from incorrectly interpreting the categories as having a meaningful order. For ordered categories such as "alcohol intake frequency" and "general health rating," where the order is relevant, these features were kept as individual non-binary columns.

Input	UKBB	AoU	Harmonization Process
**Lab (blood) test results**	Results from 32 lab (blood) tests, consistent units per test	Results from the same 32 lab tests, some with different units	Converted units found in AoU to those found in UKBB, for a given test
**Age**	Date of birth provided as yyyy-mm-dd	Date of birth provided as yyyy-mm-dd	Calculated age at first blood test recorded in the dataset
**Race / ethnicity**	Ethnic background provided as White, Mixed, Asian or Asian British, Black or Black British Chinese, or Other ethnic group	Race/ethnicity provided as Asian, Black, Hispanic, MENA, NHPI, White, or Other	White = White<br>Asian or Asian British & Chinese = Asian<br>Black or Black British = Black<br>Mixed = n/a<br>Other ethnic group = Hispanic & MENA & NHPI & Other
**Medication usage**	Field noting use of cholesterol lowering medication, blood pressure medication, or insulin	Searched dataset for drugs used to lower cholesterol or control blood pressure	Binary field noting use of any cholesterol lowering medication, and another noting use of any blood pressure medication
**Comorbidities**	Searched dataset for comorbidities common in T2D	Searched dataset for comorbidities common in T2D	Binary fields indicating comorbidities of interest
**Current smoking status**	No, only occasionally, or yes on most or all days	Not at all, on some days, or every day	No = Not at all<br>Occasionally = On some days<br>Yes on most or all days = Every day
**Smoking history**	Never, previous, or current	Combination of current smoking status and whether they have smoked at least 100 cigarettes in their entire life (yes/no)	*Defined "have smoked" as having ≥100 cigarettes in life*<br>Never = Not at all + no<br>Previous = Not at all + yes<br>Current = On some days/every day + yes
**Alcohol intake frequency**	Never, special occasions only, 1-3 times a month, 1-2 times a week, 3-4 times a week, or daily or almost daily	Never, monthly or less, 2-4 times per month, 2-3 times per week, or 4 or more times per week	Never = Never<br>Monthly or less = Special occasions only<br>2-4 times per month or 2-3 times per week = 1-3 times a month or 1-2 times a week or 3-4 times a week<br>4+ per week = Daily or almost daily
**Self-rating of overall health**	Poor, fair, good, or excellent	Poor, fair, good, very good, or excellent	Poor = Poor<br>Fair = Fair<br>Good = Good or very good<br>Excellent = Excellent

Supplemental Figure S1: Details on harmonization of the UK Biobank (UKBB) and All of Us (AoU) datasets. MENA = Middle East and North Africa; NHPI = Native Hawaiian and Pacific Islander; T2D = type 2 diabetes.

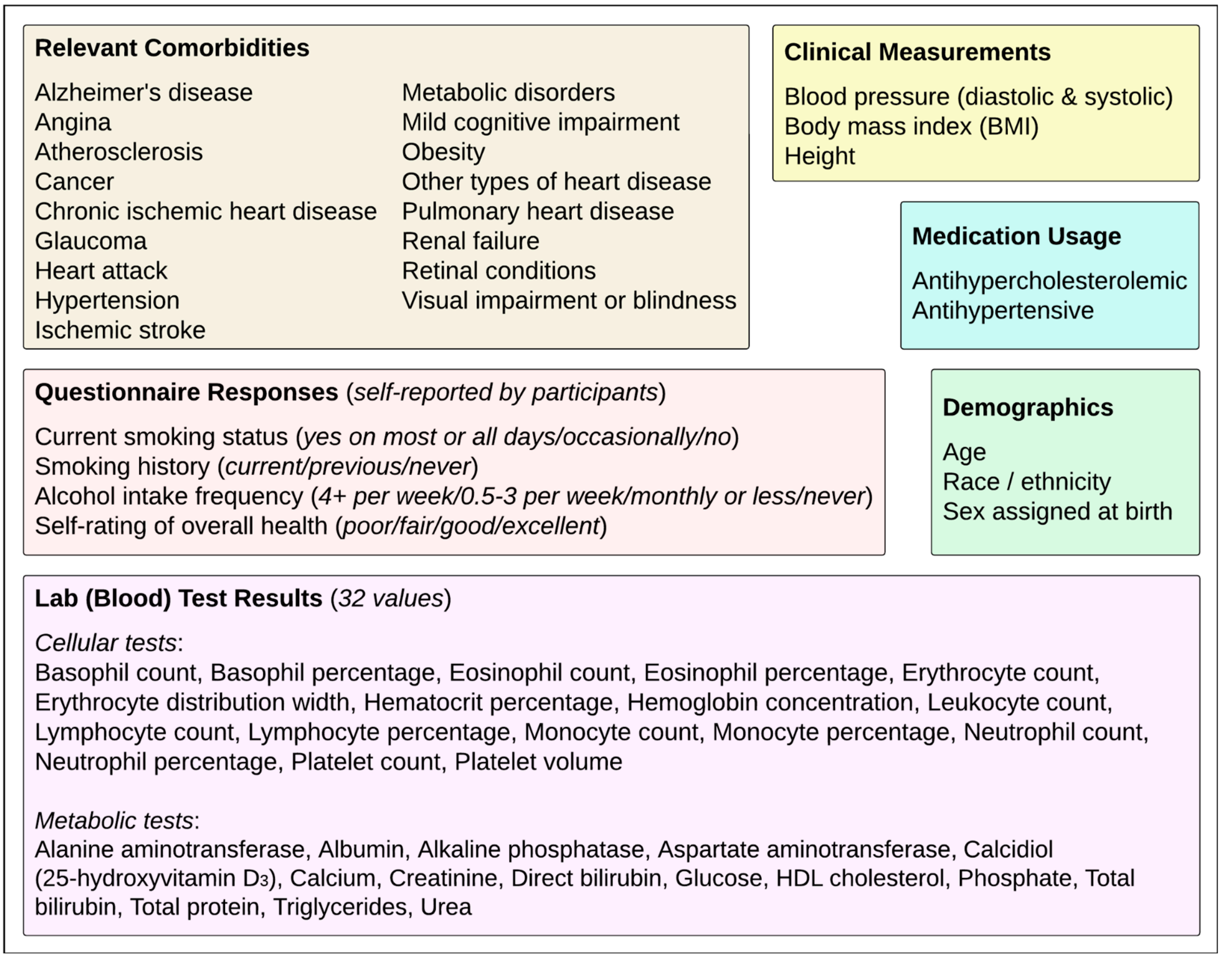

Supplemental Figure S2: Machine learning algorithm (MLA) inputs.

Supplemental Table S2: Performance metrics of three candidate ML approaches on the test sets for each of the three datasets. Each AUROC is presented as the mean of 1,000 bootstrapped AUROC values along with the 95% CI (the middle 95% of the 1000 bootstrapped values). The 95% CIs for specificity, specificity, PPV, and NPV were calculated using normal approximation.

Classifier	Performance Metric	UKBB Dataset	AoU Dataset	Combined Dataset
Random Forest	AUROC (95% CI)	0.622 (0.573 - 0.671)	0.666 (0.646 - 0.687)	0.762 (0.747 - 0.778)
	Sensitivity (95% CI)	0.655 (0.577 - 0.733)	0.997 (0.995 - 1.000)	0.965 (0.955 - 0.976)
	Specificity (95% CI)	0.489 (0.476 - 0.502)	0.035 (0.032 - 0.038)	0.238 (0.230 - 0.245)
	PPV (95% CI)	0.031 (0.025 - 0.037)	0.126 (0.120 - 0.131)	0.103 (0.097 - 0.109)
	NPV (95% CI)	0.983 (0.978 - 0.988)	0.989 (0.979 - 0.999)	0.987 (0.983 - 0.991)
KNN	AUROC (95% CI)	0.526 (0.478 - 0.578)	0.567 (0.546 - 0.589)	0.668 (0.651 - 0.685)
	Sensitivity (95% CI)	0.578 (0.547 - 0.609)	0.868 (0.860 - 0.877)	0.824 (0.816 - 0.833)
	Specificity (95% CI)	0.494 (0.489 - 0.498)	0.224 (0.220 - 0.228)	0.439 (0.435 - 0.442)
	PPV (95% CI)	0.027 (0.025 - 0.030)	0.135 (0.131 - 0.138)	0.118 (0.115 - 0.121)
	NPV (95% CI)	0.979 (0.977 - 0.981)	0.924 (0.919 - 0.929)	0.965 (0.963 - 0.967)
MLP	AUROC (95% CI)	0.554 (0.500 - 0.612)	0.563 (0.542 - 0.585)	0.669 (0.651 - 0.685)
	Sensitivity (95% CI)	0.500 (0.497 - 0.503)	0.551 (0.548 - 0.555)	0.550 (0.549 - 0.552)
	Specificity (95% CI)	0.592 (0.586 - 0.597)	0.536 (0.528 - 0.544)	0.695 (0.695 - 0.696)
	PPV (95% CI)	0.029 (0.028 - 0.029)	0.142 (0.140 - 0.143)	0.141 (0.141 - 0.142)
	NPV (95% CI)	0.980 (0.977 - 0.983)	0.895 (0.889 - 0.901)	0.944 (0.944 - 0.945)

AoU = All of Us, AUROC = area under the receiver operating characteristic, CI = confidence interval, KNN = k-nearest neighbors, ML = machine learning, MLA = machine learning algorithm, MLP = multi-layer perceptron, NPV = negative predictive value, PPV = positive predictive value, UKBB = UK Biobank.

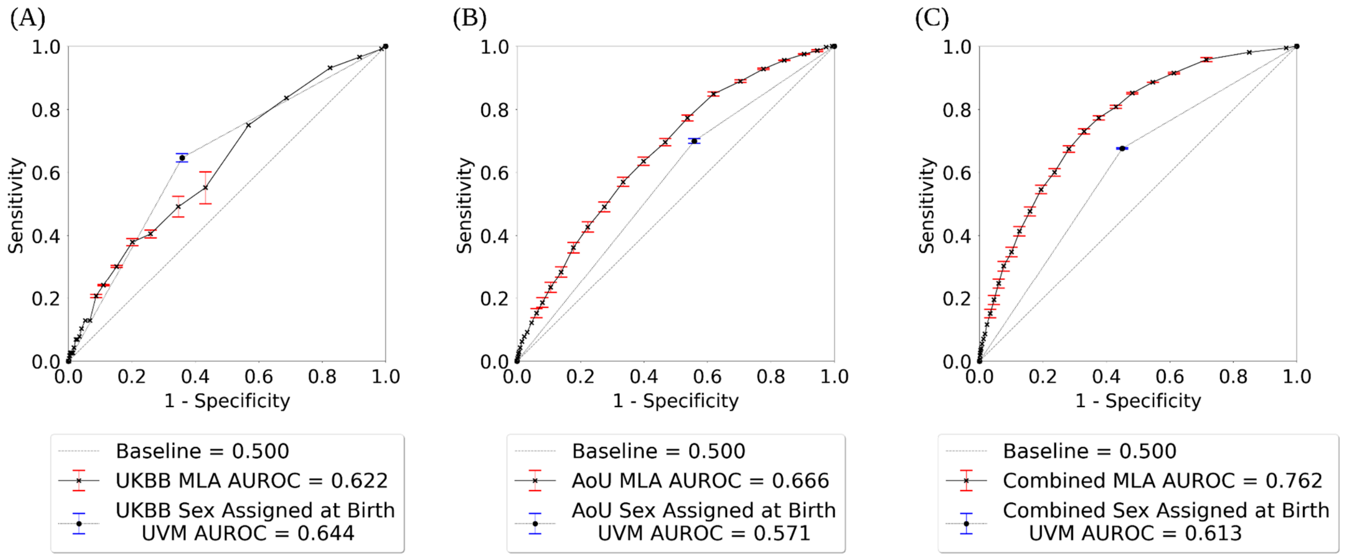

Supplemental Figure S3: The receiver operating characteristic (ROC) curves for HT vs. non-HT for each MLA and the corresponding univariate model (UVM, using only sex assigned at birth as a model input to predict HT in patients with T2D) for the (A) UKBB dataset, (B) AoU dataset, and (C) Combined dataset. Error bars represent 95% confidence intervals (CIs) for sensitivity at each operating point displayed. AoU = All of Us; HT = hypothyroidism; MLA = machine learning algorithm; UKBB = UK Biobank.

Supplemental Table S3: Performance metrics of the three UVMs, using only sex assigned at birth as a model input to predict HT status in patients with T2D, trained and tested on the same training and test sets as the corresponding multi-variable MLAs. Each AUROC is presented as the mean of 1000 bootstrapped AUROC values along with the 95% CI (the middle 95% of the 1000 bootstrapped values). The 95% CIs for specificity, specificity, PPV, and NPV were calculated using normal approximation.

**Performance Metrics**	**UKBB UVM**	**AoU UVM**	**Combined UVM**
AUROC (95% CI)	0.644 (0.603 - 0.686)	0.571 (0.552 - 0.588)	0.613 (0.596 - 0.630)
Sensitivity (95% CI)	0.647 (0.634 - 0.660)	0.699 (0.692 - 0.707)	0.676 (0.674 - 0.678)
Specificity (95% CI)	0.642 (0.633 - 0.651)	0.442 (0.439 - 0.445)	0.550 (0.550 - 0.551)
PPV (95% CI)	0.043 (0.037 - 0.049)	0.159 (0.156 - 0.162)	0.118 (0.117 - 0.118)
NPV (95% CI)	0.987 (0.980 - 0.994)	0.907 (0.904 - 0.910)	0.950 (0.950 - 0.951)

AUROC = area under the receiver operating characteristic curve; CI = confidence interval; HT = hypothyroidism; MLA = machine learning algorithm; PPV = positive predictive value; NPV = negative predictive value; T2D = type 2 diabetes; UVM = univariate model.

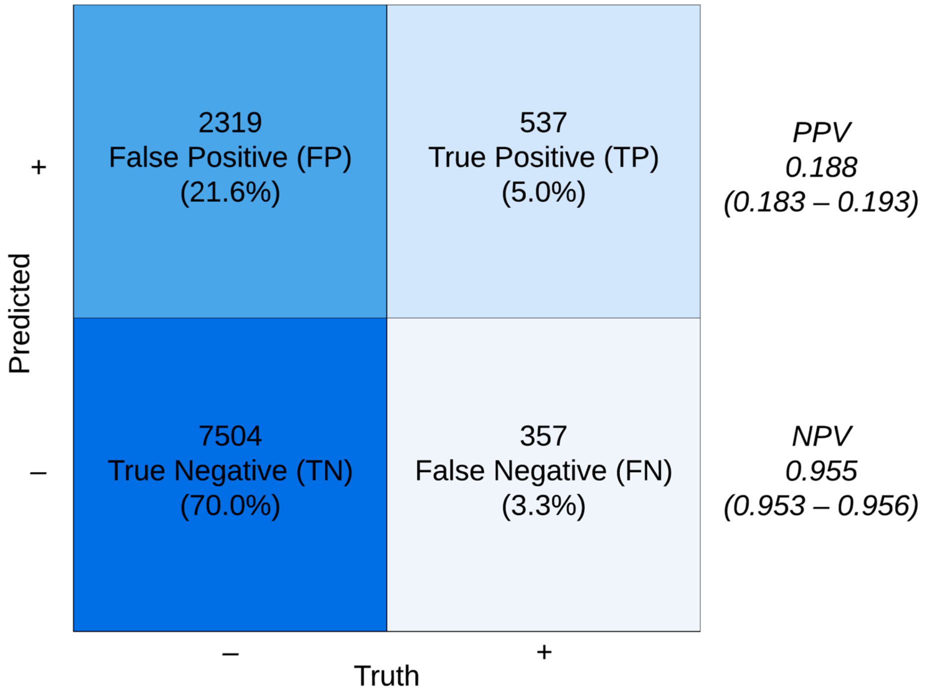

Supplemental Figure S4: Confusion matrix showcasing the ability of the Combined MLA to reliably identify a high volume of T2D patients as having a low HT risk. HT = hypothyroidism; MLA = machine learning algorithm; NPV = negative predictive value; PPV = positive predictive value; T2D = type 2 diabetes.
